# Supplementary material for: A Likelihood Approach for Real-Time Calibration of Stochastic Compartmental Epidemic Models
Source: PLoS Comput Biol. 2017 Jan 17;13(1):e1005257. doi: 10.1371/journal.pcbi.1005257 (PMC5240920; doi:10.1371/journal.pcbi.1005257)
Supplement: S1 File — (TAR.GZ) [file pcbi.1005257.s014.tar.gz › HSPH_Online-SI-Revision/IPoi09/n10-extreme/n10-extreme_BENCH2_table-4.pdf]

1	newly 3 weeks spec	1350.	1226.96	{908., 1424.}	11.9566	0.0784961	newly 3 weeks cumul	2770.	3153.29	{2115., 3986.}	30.294	0.149538	newly 5 weeks spec	1264.	994.171	{719., 1259.}	22.292	0.119877	newly 5 weeks cumul	5455.	5314.86	{4550., 5876.}	9.80542	0.0542631	newly 62 weeks cumul	8628.	8071.71	{7156., 8941.}	8.14359	0.0382178
2	newly 3 weeks spec	604.	782.515	{553., 973.}	39.3287	0.157749	newly 3 weeks cumul	1548.	2300.05	{1498., 2843.}	58.8159	0.219762	newly 5 weeks spec	678.	625.591	{368., 913.}	27.8471	0.13603	newly 5 weeks cumul	2970.	3647.53	{2861., 4303.}	31.0688	0.127184	newly 58 weeks cumul	6502.	5853.05	{4077., 7570.}	18.1271	0.0897285
3	newly 3 weeks spec	1586.	1593.99	{1140., 1853.}	15.6324	0.0791315	newly 3 weeks cumul	3187.	3365.01	{1766., 4423.}	28.3878	0.136279	newly 5 weeks spec	1325.	1321.56	{945., 1701.}	17.3798	0.0775445	newly 5 weeks cumul	6258.	6313.23	{5306., 6937.}	9.68068	0.0447536	newly 64 weeks cumul	8818.	8866.1	{8514., 9218.}	2.75477	0.012069
4	newly 3 weeks spec	888.	963.708	{804., 1111.}	19.2013	0.107837	newly 3 weeks cumul	2367.	2586.78	{1692., 3112.}	24.8728	0.135003	newly 5 weeks spec	862.	864.585	{592., 1061.}	15.7094	0.0757126	newly 5 weeks cumul	4124.	4395.49	{3785., 4938.}	15.5797	0.0818568	newly 58 weeks cumul	7204.	7933.89	{6705., 8967.}	13.5845	0.0555176
5	newly 3 weeks spec	1149.	864.522	{510., 1265.}	28.5503	0.161937	newly 3 weeks cumul	2806.	3209.41	{2305., 3964.}	23.6558	0.0973321	newly 5 weeks spec	1043.	449.13	{201., 750.}	57.6612	0.414736	newly 5 weeks cumul	5000.	4291.84	{2844., 5647.}	19.7841	0.102181	newly 61 weeks cumul	8544.	5215.64	{3250., 7290.}	39.0855	0.233177
6	newly 3 weeks spec	1137.	1124.13	{592., 1421.}	21.9536	0.117389	newly 3 weeks cumul	2504.	2874.74	{1184., 3896.}	36.7927	0.174083	newly 5 weeks spec	1175.	905.065	{434., 1352.}	30.7948	0.194218	newly 5 weeks cumul	4971.	4863.45	{3453., 5859.}	14.1842	0.0725328	newly 62 weeks cumul	8740.	6825.16	{5409., 8334.}	21.9288	0.116097
7	newly 3 weeks spec	962.	1301.6	{959., 1600.}	38.6084	0.138724	newly 3 weeks cumul	2111.	3638.27	{2260., 4455.}	73.3918	0.229409	newly 5 weeks spec	1027.	812.492	{361., 1391.}	35.9902	0.212094	newly 5 weeks cumul	4213.	5565.71	{4757., 6360.}	32.9686	0.121307	newly 63 weeks cumul	7880.	6949.9	{5316., 8400.}	15.6329	0.0785133
8	newly 3 weeks spec	963.	1028.97	{817., 1170.}	14.5102	0.0617265	newly 3 weeks cumul	2334.	3037.92	{2352., 3407.}	35.6218	0.136175	newly 5 weeks spec	978.	792.8	{532., 1100.}	23.6755	0.124888	newly 5 weeks cumul	4380.	4760.73	{4091., 5191.}	12.5556	0.052305	newly 60 weeks cumul	8557.	7278.83	{5636., 8593.}	15.7346	0.0783195
9	newly 3 weeks spec	987.	1077.13	{799., 1284.}	19.511	0.101966	newly 3 weeks cumul	2373.	3134.4	{2717., 3628.}	39.8162	0.17385	newly 5 weeks spec	932.	784.868	{448., 1101.}	24.2663	0.138925	newly 5 weeks cumul	4333.	4873.5	{3995., 5470.}	19.006	0.0944593	newly 60 weeks cumul	8041.	7157.37	{5477., 8445.}	13.6142	0.0675981
10	newly 3 weeks spec	784.	857.031	{520., 1183.}	29.843	0.126759	newly 3 weeks cumul	1916.	2861.41	{2224., 3466.}	51.5723	0.176684	newly 5 weeks spec	979.	546.656	{237., 900.}	46.16	0.317887	newly 5 weeks cumul	3879.	4112.69	{2886., 5161.}	21.9636	0.0946677	newly 60 weeks cumul	8180.	5425.84	{3328., 7491.}	33.9804	0.202968
11	newly 3 weeks spec	908.	622.966	{158., 821.}	31.3915	0.247686	newly 3 weeks cumul	2169.	1686.93	{316., 2391.}	28.1283	0.240969	newly 5 weeks spec	841.	634.207	{300., 803.}	24.589	0.16974	newly 5 weeks cumul	3950.	2964.57	{795., 3796.}	24.959	0.197769	newly 55 weeks cumul	7949.	7416.6	{5870., 8682.}	11.0547	0.0529864
12	newly 3 weeks spec	811.	839.742	{454., 1108.}	25.9178	0.131272	newly 3 weeks cumul	1803.	1985.69	{862., 2894.}	36.5654	0.173347	newly 5 weeks spec	1021.	922.242	{693., 1089.}	13.0122	0.0668978	newly 5 weeks cumul	3891.	3834.6	{2463., 4771.}	18.0329	0.0919725	newly 60 weeks cumul	7914.	7615.42	{6440., 8815.}	10.1547	0.0469053
13	newly 3 weeks spec	841.	708.571	{302., 908.}	20.6236	0.14746	newly 3 weeks cumul	2317.	1834.95	{616., 2606.}	27.2623	0.202216	newly 5 weeks spec	848.	761.889	{616., 893.}	13.1009	0.0747051	newly 5 weeks cumul	4080.	3348.37	{1706., 4143.}	18.7368	0.129137	newly 56 weeks cumul	7098.	8031.43	{7033., 8878.}	14.1207	0.0562995
14	newly 3 weeks spec	971.	1147.25	{815., 1398.}	26.0168	0.102064	newly 3 weeks cumul	2497.	3595.9	{2864., 4024.}	47.2277	0.169478	newly 5 weeks spec	946.	704.232	{371., 1109.}	33.8481	0.191423	newly 5 weeks cumul	4507.	5230.72	{4208., 5866.}	19.5776	0.0775745	newly 61 weeks cumul	7771.	6740.03	{4950., 8341.}	16.1785	0.0814656
15	newly 3 weeks spec	832.	946.544	{862., 1068.}	18.9073	0.0842309	newly 3 weeks cumul	2173.	2677.41	{2144., 3084.}	29.0531	0.12256	newly 5 weeks spec	838.	799.191	{573., 1018.}	16.0378	0.075814	newly 5 weeks cumul	3908.	4363.06	{3961., 4774.}	15.7219	0.0691417	newly 58 weeks cumul	6416.	7488.96	{6044., 8656.}	19.321	0.0756193
16	newly 3 weeks spec	1062.	993.417	{479., 1293.}	21.7383	0.13456	newly 3 weeks cumul	2353.	2265.4	{914., 3492.}	31.4958	0.184229	newly 5 weeks spec	1082.	1074.35	{795., 1263.}	14.5397	0.0709309	newly 5 weeks cumul	4527.	4431.93	{2759., 5417.}	18.3998	0.101786	newly 61 weeks cumul	8363.	8359.26	{7767., 8894.}	5.10931	0.0227221
17	newly 3 weeks spec	1033.	1357.65	{858., 1662.}	44.9285	0.191121	newly 3 weeks cumul	2323.	3313.05	{1458., 4463.}	60.7869	0.249102	newly 5 weeks spec	1116.	1035.13	{535., 1461.}	26.0952	0.134555	newly 5 weeks cumul	4627.	5638.78	{4460., 6520.}	30.6771	0.132009	newly 63 weeks cumul	8302.	7830.62	{6505., 8701.}	8.35695	0.0395762
18	newly 3 weeks spec	1151.	1165.47	{905., 1353.}	15.0994	0.078403	newly 3 weeks cumul	2756.	2869.06	{1749., 3711.}	25.6553	0.128414	newly 5 weeks spec	1119.	1059.28	{761., 1326.}	15.5641	0.0745299	newly 5 weeks cumul	5058.	5109.19	{4285., 5675.}	11.3934	0.0562817	newly 61 weeks cumul	8663.	8256.91	{7246., 9069.}	6.61128	0.0304724
19	newly 3 weeks spec	1098.	1087.61	{459., 1454.}	26.7841	0.152252	newly 3 weeks cumul	2182.	2217.78	{765., 3364.}	35.5425	0.19413	newly 5 weeks spec	1350.	1267.81	{1053., 1452.}	10.0509	0.0520251	newly 5 weeks cumul	4959.	4764.06	{2856., 5909.}	17.5418	0.0961424	newly 63 weeks cumul	8373.	8774.55	{8232., 9156.}	5.82434	0.0245439
20	newly 3 weeks spec	902.	975.111	{648., 1180.}	21.6402	0.0963987	newly 3 weeks cumul	2235.	2234.75	{1272., 3017.}	24.217	0.118223	newly 5 weeks spec	986.	1071.08	{898., 1182.}	12.6944	0.0523944	newly 5 weeks cumul	4273.	4395.22	{3380., 5087.}	14.1756	0.0642978	newly 60 weeks cumul	8476.	8637.15	{8094., 9084.}	4.18223	0.017955
21	newly 3 weeks spec	966.	825.45	{617., 986.}	15.4572	0.118686	newly 3 weeks cumul	2273.	2268.32	{1355., 2811.}	22.8956	0.152216	newly 5 weeks spec	836.	758.533	{526., 949.}	16.9179	0.0951329	newly 5 weeks cumul	4121.	3843.05	{3097., 4438.}	12.1795	0.0879861	newly 57 weeks cumul	7668.	7526.32	{6033., 8704.}	10.3336	0.0467302
22	newly 3 weeks spec	1428.	1177.13	{294., 1623.}	25.4914	0.197482	newly 3 weeks cumul	2568.	2324.07	{476., 3807.}	36.0898	0.248007	newly 5 weeks spec	1622.	1319.52	{939., 1652.}	19.514	0.116255	newly 5 weeks cumul	6049.	5023.64	{2313., 6462.}	19.1059	0.133022	newly 64 weeks cumul	9138.	8577.57	{7732., 9253.}	6.63321	0.0308243
23	newly 3 weeks spec	671.	779.604	{458., 1092.}	31.9264	0.129931	newly 3 weeks cumul	1760.	2677.91	{1925., 3262.}	56.071	0.189969	newly 5 weeks spec	667.	454.509	{221., 736.}	39.7839	0.246265	newly 5 weeks cumul	3172.	3745.08	{2580., 4708.}	27.139	0.107712	newly 59 weeks cumul	5972.	4733.21	{2928., 6354.}	24.577	0.137189
24	newly 3 weeks spec	922.	856.31	{614., 1033.}	14.3167	0.0723307	newly 3 weeks cumul	2515.	2592.	{1882., 2972.}	16.6634	0.0844744	newly 5 weeks spec	778.	692.493	{483., 950.}	21.3965	0.107074	newly 5 weeks cumul	4256.	4072.37	{3109., 4616.}	10.8003	0.053335	newly 57 weeks cumul	7579.	6889.28	{5213., 8834.}	16.0685	0.0782118
25	newly 3 weeks spec	1376.	561.861	{164., 920.}	59.1671	0.478788	newly 3 weeks cumul	2783.	1208.82	{324., 2044.}	56.6141	0.47317	newly 5 weeks spec	1327.	828.903	{496., 1040.}	37.5356	0.238555	newly 5 weeks cumul	5644.	2758.06	{1145., 4111.}	51.133	0.376498	newly 60 weeks cumul	8660.	8810.6	{8439., 9209.}	3.44319	0.0148169
26	newly 3 weeks spec	1193.	1294.42	{711., 1677.}	26.0139	0.115445	newly 3 weeks cumul	2728.	3443.02	{2264., 4406.}	38.0632	0.146379	newly 5 weeks spec	913.	870.808	{231., 1485.}	40.8965	0.234082	newly 5 weeks cumul	4845.	5465.94	{3957., 6395.}	21.003	0.0857457	newly 63 weeks cumul	6836.	6943.63	{4700., 8332.}	18.3106	0.084772
27	newly 3 weeks spec	1034.	1012.06	{479., 1296.}	22.1288	0.129436	newly 3 weeks cumul	2307.	2311.45	{914., 3330.}	31.8583	0.178976	newly 5 weeks spec	1086.	1076.51	{774., 1264.}	13.6533	0.0667148	newly 5 weeks cumul	4510.	4490.91	{2759., 5440.}	18.1024	0.0980143	newly 61 weeks cumul	8570.	8388.03	{7767., 9006.}	5.03746	0.0229146
28	newly 3 weeks spec	1424.	1526.79	{1108., 1811.}	19.2851	0.0848565	newly 3 weeks cumul	2886.	4297.9	{3240., 4996.}	54.8959	0.193011	newly 5 weeks spec	1362.	874.535	{467., 1330.}	40.1357	0.254116	newly 5 weeks cumul	5761.	6409.15	{5446., 6962.}	15.6636	0.0648516	newly 64 weeks cumul	8981.	7839.68	{6537., 9006.}	13.1335	0.0662175
29	newly 3 weeks spec	1269.	607.015	{195., 971.}	52.1659	0.417205	newly 3 weeks cumul	2814.	1377.41	{368., 2420.}	51.0817	0.434791	newly 5 weeks spec	1154.	818.364	{481., 991.}	29.0846	0.190543	newly 5 weeks cumul	5251.	2925.67	{1161., 4342.}	44.2836	0.328509	newly 59 weeks cumul	8475.	8829.2	{8148., 9208.}	5.49012	0.0232228
30	newly 3 weeks spec	975.	945.826	{636., 1114.}	14.6667	0.0973157	newly 3 weeks cumul	2275.	2523.14	{1430., 3134.}	29.7028	0.156251	newly 5 weeks spec	882.	863.652	{653., 1061.}	14.6801	0.071464	newly 5 weeks cumul	4197.	4325.9	{3516., 4958.}	15.164	0.0826196	newly 59 weeks cumul	7393.	7943.62	{6510., 8990.}	11.3697	0.0469941
31	newly 3 weeks spec	1130.	1273.2	{856., 1526.}	24.1413	0.10151	newly 3 weeks cumul	2628.	3381.14	{2017., 4193.}	41.037	0.161971	newly 5 weeks spec	1066.	907.656	{402., 1369.}	29.2888	0.16275	newly 5 weeks cumul	4862.	5440.27	{4106., 6210.}	17.8126	0.0731994	newly 62 weeks cumul	8496.	7220.44	{5638., 8544.}	15.5912	0.0786034
32	newly 3 weeks spec	826.	618.133	{149., 800.}	25.7466	0.199664	newly 3 weeks cumul	2183.	1668.97	{316., 2374.}	28.0363	0.229803	newly 5 weeks spec	784.	634.133	{300., 789.}	19.7917	0.124952	newly 5 weeks cumul	3849.	2940.1	{795., 3677.}	23.7672	0.175688	newly 54 weeks cumul	7242.	7438.37	{6030., 8679.}	11.9217	0.0517016
33	newly 3 weeks spec	1646.	1145.09	{840., 1402.}	30.4322	0.180314	newly 3 weeks cumul	3764.	2531.23	{1476., 3636.}	33.4113	0.21522	newly 5 weeks spec	1179.	1201.54	{901., 1406.}	13.4715	0.0611498	newly 5 weeks cumul	6592.	5004.21	{3988., 5815.}	24.0866	0.132263	newly 62 weeks cumul	8743.	8656.2	{7905., 9049.}	3.49667	0.0156018
34	newly 3 weeks spec	1346.	921.652	{398., 1807.}	43.4492	0.269451	newly 3 weeks cumul	2950.	3824.09	{2472., 4725.}	35.5696	0.130393	newly 5 weeks spec	1080.	258.217	{77., 632.}	76.091	0.751057	newly 5 weeks cumul	5397.	4601.09	{2768., 6264.}	22.4118	0.117817	newly 64 weeks cumul	7875.	4821.61	{2871., 6827.}	39.1625	0.235416
35	newly 3 weeks spec	787.	846.9	{505., 1133.}	27.5168	0.120376	newly 3 weeks cumul	2046.	2785.71	{2031., 3336.}	41.1702	0.150765	newly 5 weeks spec	782.	571.4	{265., 936.}	35.97	0.218339	newly 5 weeks cumul	3686.	4068.17	{2769., 5072.}	23.5059	0.099189	newly 59 weeks cumul	7161.	5677.29	{3469., 7873.}	25.7003	0.144189
36	newly 3 weeks spec	900.	708.71	{302., 908.}	21.7814	0.159508	newly 3 weeks cumul	2289.	1843.29	{616., 2616.}	27.5215	0.202793	newly 5 weeks spec	946.	756.21	{543., 893.}	20.0726	0.113636	newly 5 weeks cumul	4201.	3347.11	{1706., 4143.}	20.4694	0.139821	newly 57 weeks cumul	8382.	8000.48	{6873., 8878.}	7.92943	0.0365079
37	newly 3 weeks spec	1140.	922.877	{594., 1168.}	20.1633	0.148769	newly 3 weeks cumul	2692.	2219.	{1071., 3286.}	28.2735	0.200132	newly 5 weeks spec	1097.	990.769	{740., 1169.}	13.8658	0.0790592	newly 5 weeks cumul	4963.	4221.32	{3111., 5088.}	16.1066	0.109592	newly 60 weeks cumul	8552.	8540.74	{7537., 9216.}	5.79298	0.0256434
38	newly 3 weeks spec	1819.	880.882	{338., 1367.}	51.5733	0.420663	newly 3 weeks cumul	3439.	1707.18	{559., 3018.}	50.3583	0.413118	newly 5 weeks spec	1286.	1251.29	{1047., 1435.}	11.4331	0.111207	newly 5 weeks cumul	6558.	4110.78	{2271., 5600.}	37.3166	0.27262	newly 63 weeks cumul	8641.	8774.65	{8489., 9266.}	5.46642	0.0704676
39	newly 3 weeks spec	1549.	1323.1	{655., 1640.}	18.1226	0.137725	newly 3 weeks cumul	3391.	2914.62	{1084., 4253.}	29.0151	0.204929	newly 5 weeks spec	1272.	1237.59	{927., 1594.}	17.4694	0.0854886	newly 5 weeks cumul	6224.	5545.06	{3957., 6481.}	13.7358	0.0900067	newly 63 weeks cumul	8953.	8755.06	{8240., 9208.}	3.85613	0.0171969
40	newly 3 weeks spec	954.	1038.02	{738., 1261.}	20.4646	0.087347	newly 3 weeks cumul	2231.	3038.55	{2481., 3596.}	41.5373	0.155932	newly 5 weeks spec	887.	704.518	{371., 1060.}	30.9571	0.175786	newly 5 weeks cumul	4133.	4635.38	{3706., 5279.}	17.7245	0.0723647	newly 60 weeks cumul	7710.	6174.93	{4484., 7723.}	20.4868	0.107013
41	newly 3 weeks spec	1055.	955.324	{804., 1116.}	12.476	0.0708789	newly 3 weeks cumul	2675.	2563.14	{1736., 3124.}	18.9987	0.106756	newly 5 weeks spec	964.	870.797	{588., 1070.}	16.6942	0.0834951	newly 5 weeks cumul	4723.	4376.93	{3830., 4930.}	9.61625	0.052847	newly 59 weeks cumul	8244.	7931.36	{6289., 9004.}	9.46979	0.0441154
42	newly 3 weeks spec	812.	710.081	{511., 857.}	15.4239	0.119137	newly 3 weeks cumul	2241.	1997.23	{1113., 2478.}	18.7661	0.146036	newly 5 weeks spec	727.	669.371	{472., 834.}	16.819	0.106006	newly 5 weeks cumul	3835.	3370.77	{2456., 3937.}	13.2616	0.104122	newly 54 weeks cumul	7356.	7113.47	{5452., 8481.}	12.8206	0.0591406
43	newly 3 weeks spec	1345.	982.647	{343., 1538.}	32.482	0.225816	newly 3 weeks cumul	2361.	1842.21	{524., 3275.}	37.8815	0.253924	newly 5 weeks spec	1603.	1376.91	{1110., 1567.}	14.2674	0.0743441	newly 5 weeks cumul	5687.	4502.53	{2508., 5960.}	22.9838	0.138092	newly 64 weeks cumul	8914.	8860.63	{8388., 9251.}	3.31518	0.0148852
44	newly 3 weeks spec	783.	643.984	{350., 840.}	21.9627	0.177378	newly 3 weeks cumul	2116.	1735.	{731., 2463.}	29.0133	0.228604	newly 5 weeks spec	810.	675.54	{548., 817.}	17.7288	0.116655	newly 5 weeks cumul	3802.	3094.78	{1870., 3939.}	19.9456	0.154956	newly 55 weeks cumul	7747.	7583.25	{6321., 8672.}	10.3729	0.046732
45	newly 3 weeks spec	892.	1019.45	{793., 1176.}	20.0721	0.0812446	newly 3 weeks cumul	2335.	2756.74	{1892., 3331.}	26.4991	0.106069	newly 5 weeks spec	873.	857.072	{502., 1180.}	24.7041	0.121153	newly 5 weeks cumul	4172.	4583.72	{3717., 5122.}	14.3812	0.0595369	newly 59 weeks cumul	7795.	7224.57	{5237., 8525.}	12.6131	0.0633694
46	newly 3 weeks spec	811.	584.213	{337., 900.}	31.1961	0.179726	newly 3 weeks cumul	2110.	2005.41	{1303., 2644.}	20.2553	0.0965969	newly 5 weeks spec	861.	405.893	{209., 717.}	53.5455	0.373546	newly 5 weeks cumul	3857.	2904.32	{1771., 4110.}	27.3653	0.153149	newly 57 weeks cumul	7935.	4349.89	{2421., 7034.}	45.76	0.294612
47	newly 3 weeks spec	1061.	656.492	{195., 984.}	38.1252	0.31387	newly 3 weeks cumul	2374.	1509.83	{381., 2651.}	40.5524	0.34501	newly 5 weeks spec	1104.	835.032	{548., 1001.}	24.3631	0.178215	newly 5 weeks cumul	4682.	3115.6	{1258., 4477.}	33.4632	0.263223	newly 59 weeks cumul	8780.	8742.51	{8060., 9180.}	3.70467	0.0163685
48	newly 3 weeks spec	1085.	1220.55	{881., 1435.}	23.9411	0.121362	newly 3 weeks cumul	2381.	2885.66	{1560., 3889.}	37.956	0.172805	newly 5 weeks spec	1180.	1078.34	{660., 1412.}	19.422	0.0998652	newly 5 weeks cumul	4857.	5198.79	{4217., 5890.}	16.602	0.0836328	newly 62 weeks cumul	8650.	8093.28	{6940., 8942.}	7.86921	0.037461
49	newly 3 weeks spec	1202.	1226.38	{589., 1538.}	25.4069	0.13767	newly 3 weeks cumul	2426.	2686.17	{981., 3893.}	39.4769	0.195797	newly 5 weeks spec	1386.	1211.61	{877., 1478.}	15.8741	0.0799267	newly 5 weeks cumul	5269.	5211.58	{3500., 6203.}	16.3829	0.0839709	newly 63 weeks cumul	8545.	8476.51	{7466., 9139.}	6.07152	0.0271763
50	newly 3 weeks spec	1062.	810.391	{399., 1112.}	26.6758	0.182619	newly 3 weeks cumul	2104.	1777.3	{752., 2749.}	33.3153	0.211035	newly 5 weeks spec	1231.	1033.69	{869., 1181.}	16.0286	0.0879804	newly 5 weeks cumul	4630.	3777.11	{2317., 4840.}	21.3968	0.134122	newly 61 weeks cumul	8721.	8565.05	{7845., 9165.}	4.36429	0.0195079
